# A Public Health Approach to Address the Mental Health Burden of Youth in Situations of Political Violence and Humanitarian Emergencies

**DOI:** 10.1007/s11920-015-0590-0

**Published:** 2015-05-30

**Authors:** Joop T. V. M. de Jong, Lidewyde H. Berckmoes, Brandon A. Kohrt, Suzan J. Song, Wietse A. Tol, Ria Reis

**Affiliations:** Amsterdam Institute of Social Science Research (AISSR) University of Amsterdam, Nieuwe Achtergracht 166, 1018WV Amsterdam, The Netherlands; Boston University School of Medicine, Boston, MA USA; Duke Global Health Institute, Department of Psychiatry and Behavioral Sciences, Duke University, Durham, NC USA; Department of Mental Health, Johns Hopkins Bloomberg School of Public Health, Baltimore, MD USA; Department of Public Health & Primary Care, Leiden University Medical Center, Leiden, The Netherlands; The Children’s Institute, School of Child and Adolescent Health, University of Cape Town, Cape Town, South Africa

**Keywords:** Public health, Youth, Humanitarian emergencies, Resilience, Mental health, Syndemics

## Abstract

This paper describes how socio-ecological theory and a syndemic health systems and public health approach may help address the plight of youth in situations of political violence and humanitarian emergencies. We describe the treatment gap caused by discrepancies in epidemiological prevalence rates, individual and family needs, and available human and material resources. We propose four strategies to develop a participatory public health approach for these youth, based on principles of equity, feasibility, and a balance between prevention and treatment. The first strategy uses ecological and transgenerational resilience as a theoretical framework to facilitate a systems approach to the plight of youth and families. This theoretical base helps to engage health care professionals in a multisectoral analysis and a collaborative public health strategy. The second strategy is to translate pre-program assessment into mental health and psychosocial support (MHPSS) priorities. Defining priorities helps to develop programs and policies that align with preventive and curative interventions in multiple tiers of the public health system. The third is a realistic budgetary framework as a condition for the development of sustainable institutional capacity including a monitoring system. The fourth strategy is to direct research to address the knowledge gap about effective practices for youth mental health in humanitarian settings.

## Introduction

Public health is defined as “the science and art of preventing disease, prolonging life and promoting health through the organized efforts and informed choices of society, organizations (public and private), communities and individuals.” WHO’s Alma Ata Declaration, in 1978, underlined the importance of comprehensive horizontal primary health care (PHC) that also addresses social inequity in health and health services as a vehicle to provide promotive, preventive, curative, and rehabilitative services. However, in the 1980s, the focus on social determinants was abandoned for “selective” PHC, as countries invested in vertical programs on diseases and issues elected for reasons of prevalence, morbidity, mortality, and the feasibility for control. This approach affected children under five and women of childbearing age with programs aimed at monitoring growth, oral rehydration therapy, breast-feeding, immunization, family planning, food supplements, and female education [1, 2]. The move away from a comprehensive PHC approach was further exacerbated by the imposition of structural adjustment policies in the 1980s and 1990s, which forced “health sector reform” based on market principles, economic efficiency, and cost-effectiveness. These “reforms” further undermined many countries’ capacity to support health system development resulting in a return to vertical programs, fragmentation of health services, neglect of social determinants of health, and an erosion of community health infrastructures and intersectoral or multisectoral collaboration, i.e., the involvement of different government ministries such as health, education, and the economic sectors. “Global Health Initiatives” fit this selective focus since they are meant to address specific issues of the global health agenda, for example, immunization, AIDS, tuberculosis, and malaria. The global mental health movement, albeit inspiring, does not escape from this inherently selective approach.

In the time of Alma Ata, a systems approach similar to the one mentioned above was also emerging in child psychology and psychiatry. Socio-ecological models were developed, and Bronfenbrenner’s ecological framework for human development [3] became particularly influential and was later adopted by the US Centers for Disease Control as their prevention framework [4]. Within the newly emerging discipline of medical anthropology, criticism of the biomedical neglect of the socio-genetic and political dimensions of disease culminated in the 1990s in Singer’s innovative approach, syndemic theory, to study and manage disease interactions in social contexts of inequality and health disparity [5, 6]. Syndemic theory built on insights that disease critically interacts within a socio-political context and offers an alternative to the traditional biomedical approach that isolates, studies, and treats diseases, including mental disorders, as if they are distinct entities. In biomedicine, the simultaneous presence of more than one disease or disorder is perceived as a comorbidity, which creates problems of diagnostic overlap and nosological boundary issues. In contrast, syndemics refers to a set of health problems involving two or more afflictions interacting synergistically that are linked and contribute to an excess disease burden in a specific population [7•, 8].

To understand and address interacting determinants during human-made violence and natural disasters and their effect on young people, a call has been raised for a public mental health approach that combines the strengths of both horizontal and vertical health system approaches [9, 10]. WHO (2007) [11] defines a health system as “all activities whose primary purpose is to promote, restore, or maintain health.” For the purpose of our paper, it is relevant to recognize that all professionals in the field of child and adolescent mental health function in a broader health system, which includes formal health stakeholders such as nurses, social workers, psychologists and psychiatrists, as well as informal stakeholders such as traditional and religious healers, self-help groups, and family and community-level social support.

In this paper, we develop a public health model that combines systemic approaches as captured in socio-ecological models and syndemic theory to address the plight of youth in situations of political violence and humanitarian emergencies. We propose that professionals and stakeholders in the field of mental health shift their attention to create both a comprehensive and diversified public health approach for this population with an emphasis on community-based processes and prevention.

We build our argument as follows. We first show that a huge treatment gap exists, which is caused by discrepancies in epidemiological prevalence rates, individual and family needs, and available human and material resources. We argue that the current predominant use of a clinical psychotherapeutic model to address massive trauma in disaster situations is not likely to be successful. We then identify several strategies to develop a public health approach for these youth based on principles of equity, feasibility, participatory research, and a balance between prevention and care. Our argument is rooted in a number of systematic reviews that have recently explored children and adolescents in situations of humanitarian emergencies [12–15, 16•]. Finally, we make recommendations for integrating the results of these studies in a horizontal public health framework to mitigate the plight of youth in situations of humanitarian emergencies and of post-conflict reconstruction. We identify several major research domains that still require investigation to alleviate the massive distress of these children and adolescents, commonly referred to as youth in this paper.

## Violence and the Burden of Traumatic Stress on Children, Youth, and Families

The WHO distinguishes three broad categories of violence: self-directed violence, interpersonal violence, and collective violence. Political violence belongs to the category of collective violence and includes war and violent conflicts, state violence, terrorist acts, and mob violence. The ratio of collective violence in low- and middle-income countries (LMIC) versus high-income countries (HIC) is 10 to 1. Political violence particularly affects LMIC: a large majority of the 33 armed conflicts recorded in 2013 took place in low-income settings, and generally in Asia (39 %) and Africa (39 %) [17]. Similarly, natural disasters (which can be subsumed with armed conflict under the broad category of humanitarian crises) disproportionally affect LMIC. Five of the top 10 countries for disaster mortality in 2013 were LMIC, accounting for over 88 % of globally reported disaster mortality in the same year [18]. For interpersonal violence, both in HIC and LMIC, one in every 2–3 women will be the victim of interpersonal violence in their lifetime including rape, sexual assault, or intimate partner violence [19–21]. Exposure to this violence and trauma in childhood or adolescence may be detrimental to mental health and disrupt development in cognitive, emotional, and social domains. This delay can lead to adverse mental health, poor educational outcomes, and functional changes in brain regions associated with learning and memory [22–24]. Children who have experienced trauma are also at higher risk for longitudinal changes in emotional functioning, including heightened attention to anger and other potential threats, difficulties regulating emotion, elevated emotional and physiological reactions to stress, and heightened neural responses to threatening stimuli in regions involved in emotional processing [25–27]. Exposure to trauma in childhood predicts life-long problems in social relationships, including a difficulty trusting others, poor-quality relationships, and an elevated risk of re-victimization and developing a variety of chronic physical health problems [28, 29]. A 22-year-follow-up study in Jamaica showed that early psychosocial intervention in children with growth retardation benefited adult educational attainment and psychological functioning and reduced violent behavior. Reductions in violent behavior are extremely important given the high levels of violence in many LMIC [30]. Such long-term studies may also help determine whether these types of early intervention programs affect transgenerational transmission of violence.

Adolescence carries the highest risk for exposure to traumatic life experiences, including interpersonal violence (e.g., physical assault by non-family members, acting-out risk behavior), rape, sexual assault, injuries, accidents, and social networking events; in conflict zones, these events often involve weapons [31]. Mental health problems are common in children and adolescents exposed to trauma, since about 8–10 % develop PTSD and an even higher proportion develop other psychopathology, including behavior problems, anxiety disorders, major depression, and substance use disorders [27]. This is of concern because although approximately 50–60 % of adults with PTSD recover within 2 years, a substantial minority develops chronic PTSD [32, 33].

Exposure to violence and concomitant mental health consequences present a profound public health burden and play a pivotal role in the syndemics of war [c.f. 7]. Individuals who develop PTSD often experience impaired role functioning and reduced life-course opportunities, including poor educational attainment, unemployment, marital instability, and disability [34, 35]. The likelihood of experiencing particular types of violence and trauma varies by sex, age, ethnicity, and sexual orientation and is often increased in humanitarian crises.

## The Discrepancy Between Treatment Needs and Services

Children and adolescents constitute approximately 50 % of the population in most LMIC. Although mental health problems affect 10–20 % of children and adolescents worldwide, their mental health needs are generally neglected by government health systems, especially in LMIC [36–38]. A study conducted in 42 LMIC showed that the median 1-year prevalence for children and adolescents treated for mental health problems was 159 per 100,000 population compared to a treated prevalence of 664 per 100,000 for the adult population [39]. In post-conflict Sierra Leone, the treatment gap for youth was estimated to be over 99 % [40]. There are a number of explanations for this gap, including a weak health system due to faulty government commitment and a lack of human and financial resources. Furthermore, mental health resources are often inequitably distributed among and within countries. Evidence suggests that most mental health resources are located in or near large cities and many of these resources are heavily focused on institutional care rather than community-based care [35, 41–43]. Natural disasters and armed conflicts tend to occur in a geographic belt stretching from Southeast Asia over the Middle East, the African Great Lakes area and West Africa to the Caribbean and Central America [44]. The WHO Atlas (2011) [45] shows that these geographic areas have the lowest concentration of child and adolescent mental health resources. For example, the majority of sub-Saharan countries have no child psychiatrists and no (or only a few) psychologists or other mental health practitioners trained in child and adolescent mental health. Another service-provider factor aggravating the treatment gap is the few existing mental health workers’ preference to focus on treatment instead of prevention and to work in urban areas instead of areas with high concentrations of people affected by war or other disasters. Additional factors explaining the treatment gap are the limited applicability of evidence-based knowledge from HIC in resource-restrained settings and the local health workers and population’s lack of awareness of the treatability of mental disorders [46]. Reviews have demonstrated that the evidence base for effective mental health interventions for youth in LMIC (specifically those in areas of armed conflict) is slowly accruing [14, 15, 36, 38]. Yet, a systematic review by Jordans et al. [12] showed a lack of rigorous studies and moderate effect sizes in controlled studies of this subject. The authors also point to methodological flaws and the need for a paradigm shift away from tertiary (mainly PTSD-focused) research to primary care, the major theme of this paper. A meta-analysis of a range of mental health and psychosocial support interventions in humanitarian settings found that overall, intervention benefits internalizing (depressive/anxiety symptoms) symptoms, but not PTSD symptoms [14, 15]. Generally, there is a gap between research and practice, since there is evidence for interventions that are not commonly implemented in practice (i.e., specialized psychotherapeutic treatment of PTSD), and the most commonly implemented interventions have not been rigorously evaluated (i.e., generic individual and family counseling, child-friendly spaces, sports, and recreational activities) [14]. A consensus-based research agenda for mental health and psychosocial support in humanitarian settings specifically highlighted child- and adolescent-research questions related to family- and school-based interventions, as well as the importance of research on protective factors [15]. Thus, additional research is needed to develop a wide range of evidence-based primary care and prevention-oriented interventions, a theme we elaborate further on.

Interventions should ideally fit multiple tiers of a public health system and be sufficiently cost-effective for large youth populations in various socio-cultural contexts. To make this feasible, governments must invest more of their health budgets toward mental health and trauma-informed services, reallocate funds from residential to primary care, and ensure an equitable and sustainable distribution of these resources. We distinguish four strategies that should be addressed to develop a public health approach for youth in disaster situations based on the principles of equity, feasibility, and a balance between prevention and care.

## Four Strategies to Develop a Public Health Approach for Youth and Families in Situations of Political Violence and Humanitarian Emergencies

### Ecological and Transgenerational Resilience as a Theoretical Foundation to Facilitate a Systems Approach to the Plight of Children and Families

Many mental health professionals working with young people have been trained in systemic family interventions. Therefore, it is astounding that there has only been a quite recent paradigm shift from resilience as an individual trait to the concept of ecological resilience. Resilience (i.e., good mental health despite exposure to significant adversity) can now be seen as critically determined by the social context [9, 10, 47–49, 50•]. This is highly relevant for youth living in (post)conflict zones. Core values for many in Africa and Asia are related to system variables beyond the level of the individual child: the family, interdependency and socio-centrism, conformity to social expectations, avoiding shame and dishonor, respect for elders and ancestors, placing other’s needs ahead of one’s own, and reciprocity cf [51]. In Nepal, a socio-ecological approach elucidated push and pull factors for youths’ participation in armed conflict, including paradoxical findings such as high rates of youths’ voluntary participation in armed groups [52]. Moreover, ecologically informed statistical models revealed that 15–20 % of variance in mental health outcomes is explained by family and community-level processes, above and beyond individual child factors [52, 53]. In Burundi, Berckmoes [54] uses the concept of “elusive tactics” in post-war context to refer to practices that allow youth to navigate their post-war living conditions by being flexible, challenging the powers that constrain everyday life, while reiterating mistrustful social relations. Unfortunately, most trauma-related observational studies and interventions referring to ecological processes are limited to treatment and measurement only at the individual level [55].

In addition to an ecological view of resilience, it is equally important to use a transgenerational perspective [56–60]. Witnessing or experiencing violence or trauma may translate into children and youth also enacting violence. Furthermore, the family and community’s roles and approaches to raising children can be significant for mediating and moderating community violence. A transgenerational perspective facilitates the identification of mechanisms that help protect against or form a risk for the transmission of adversity at various ecological levels and from one generation to the next. For example, in the African Great Lakes region, children of former child soldiers had significantly more conduct problems, worse coping skills, and felt less connected to their community, siblings, and family than other children in the community [61]. A follow-up qualitative study of former child soldiers and matched civilians evaluated how intergenerational stress might be passed from former child soldiers to their children. The study found three main ways stress was transmitted: parental discipline shaped by the rebel experience, severe parental emotional distress, and the community transmission of stress including (affiliated) stigma [60]. Therefore, we propose to add a transgenerational perspective to the concept of ecological resilience.

Focusing more explicitly on socio-ecological and transgenerational perspectives has implications for our field of mental health. It may stimulate research into the dynamic relationships between population groups and across generations in relation to their socio-ecological contexts—critical knowledge to inform intervention development. It may help us to understand the syndemics of poverty resulting from the destruction of assets and livelihoods, malnourishment and disease, and the attendant effects on children’s cognitive development, as well as the breakdown of supportive microstructures due to parental death and distrust, for example. In addition, this focus urges us to mobilize forces to mitigate the transgenerational transmission of violence and to bridge the treatment gap by revitalizing a multisectoral and community-based approach to public health. For example, research may be directed at further understanding how collaboration between multiple sectors can be established, including the economic sector (for income generation among the poor, such as former child soldiers and combatants), the social sector (as a safety net for the most vulnerable, such as internally displaced and refugee families), the educational sector (for children and youth to reduce violent experiences in schools and neighborhoods), the legal sector and women’s organizations (for appropriate governance and to address human rights violations and family violence), consumer groups (for self-help groups; for example, in cases of gender-based violence), and insurance and other companies (for public-private partnerships in public health) [62, 63]. From an ecological perspective, multisectoral collaboration would strengthen mesostructures, the relationships between teachers, parents, and other adults in microstructures relevant to children. This implies breaking down the vertical organization of sectors and stimulating horizontal cooperation in favor of children’s well-being, particularly in education and health.

### Translating a Pre-program Assessment Into Mental Health and Psychosocial Support Priorities and Policies

Establishing a mental health and psychosocial support (MHPSS) program for youth requires a pre-program assessment of the context (including political, social, cultural, gender, epidemiological, legal, governance, and institutional issues) in which both existing resources and challenges are identified [64•]. It is paramount to maximize the participation of youth to share ownership and have insight into their needs and concerns. Assessments should use mixed methods and should be repeated to adapt the program to changing community and family needs and the dynamics of recovery. An assessment includes the following components from the microlevel (youth, families) to the macrolevel (government, UN): (1) youth’s experiences in the emergency; (2) threats to mental health and psychosocial well-being, protection issues, and an inventory of risk, protective, and resilience factors; (3) mapping human resource capacity in the (N)GO (non-governmental) sectors; (4) identifying community responses, social support, coping, and life skills; (5) identifying stigma, taboos, and shame; (6) asking community leaders and traditional healers about cultural, religious, spiritual supports, rituals, and local interpretations of the causes and effects of the emergency; and (7) determining agency, interagency, and stakeholders’ plans for an MHPSS response; [44, 64•, 65, 66]. For example, in Nepal, a child-participatory approach was used not only to develop intervention contents, but also to design strategies for evaluation that could be conducted, interpreted, and disseminated by youth stakeholders [67].

The second step is to translate the information generated by the assessment into program priorities, goals, objectives, interventions, and policy. De Jong [46, 66] previously described a framework to enable program prioritization using the following public (mental) health criteria: (1) locally perceived needs and concerns; (2) prevalence and incidence; (3) severity of problems and disorders (in terms of disability, physical and psychiatric comorbidity, quality of life, days out of role, stigma); (4) treatability and feasibility (number of primary care staff, mental health professionals, healers, emHealth, community lay workers, befrienders); (5) expertise, knowledge and availability of practitioners; (6) ethical applicability (e.g., whether primary care workers can provide psychotherapy with or without pharmacotherapy); (7) political acceptability (e.g., in managing human rights violations); (8) cultural sensitivity; (9) program sustainability, and (10) cost-effectiveness. Kohrt et al. [68] have developed a framework grounded in child development for designing interventions that can incorporate De Jong’s public mental health criteria.

The third step continues the process of a step-wise policy formulation by determining: (1) the mix of preventive and curative interventions in a multilayered program that will lead to the best results, and distinguishing universal, selective and indicated preventive interventions; (2) the tier of the public health care system that is the appropriate intervention level; and (3) how to achieve optimal accessibility, equity, and quality across population groups including youth and (other) vulnerable groups, strong evidence bases and good practices and dissemination. This step-wise policy formulation results in a matrix that combines universal, selective, and indicated interventions at the (inter)national, community, family, and individual levels. For example, in the (inter)national arena, universal prevention may aim at laws to prohibit child soldier recruitment, the involvement of peace-keeping and peace-enforcing troops, or peace agreements to prevent the reemergence of political violence. On the community level, promotion of reconciliation and mediation skills among ethnic or religious groups may be regarded as selective prevention. Similarly, on the community level, classroom-based school interventions may be seen as selective interventions for distressed youth. Within the microstructure of the family, universal preventive interventions may address parental need for insight into their children’s developmental needs and non-violent disciplining skills. Selective interventions may be offered for vulnerable households (e.g., child-headed, one-parent), and indicated interventions for families with an identified problem (e.g., chronically ill or substance-abusing parent, disabled children, established abuse). On and individual level, an indicated treatment program may aim to prevent the progression of disease and disability, for example, the standard treatment for PTSD with trauma-focused CBT or other trauma-related mental health problems.

### Budgetary Framework, Sustainability, and Monitoring

It is obvious that any sustainable program needs a sound budgetary framework including allocation of resources to subnational levels and non-state actors. Such a framework is a condition for the development of sustainable institutional capacity, which should include a monitoring system. As we have seen to date, governments are often reticent to allocate sufficient funding to mental health services in community settings, instead, they often focus funding on psychiatric hospitals in urban settings. At the same time, humanitarian actors frequently operate independently of existing national systems, such as health, education, and social services. Doing so threatens the sustainability of mental health programs implemented as part of humanitarian responses and early recovery [14]. For example, one of the WHO’s recent initiatives, ‘Building back better’, aims to improve the lives of people through mental health reform after emergencies. However, a major challenge is promoting ways in which mental health programs in humanitarian settings can establish longer-term mental health system-building in countries where such systems are commonly not available [13]. This is especially true in LMIC where systems are already poor prior to an emergency and further impoverished by political violence with a concomitant decline of the public health structure.

For the purpose of this paper, we believe it is most appropriate to define sustainability as the potential to develop high-quality interventions that are integrated in local communities and services and maintained over time. Sustainability should be a priority from the very beginning of a program, and depends on quality, volume, and timing. If the quality of MHPSS support or its effectiveness is insufficient, interventions will dissipate over time and hence not be sustainable. Volume of support is related to building sufficient human resource and institutional capacity to cover a population. An intervention must reach new cohorts while taking human resource attrition into account. Timing of support should focus on whether the program can be maintained and developed over longer periods, preferably generations, and includes an adequate exit strategy. Factors promoting sustainability are: (1) a rational project design; (2) local ownership by youth and other stakeholders; (3) adequate management and cost-effectiveness; (4) a small dependency gap in terms of external funding; (5) attention to gender issues, age groups, and local health care concerns; (6) integration of local services and collaboration with local institutions such as universities, the government, and other sectors; (7) continued upgrading of the quality of human resources and provision of psychotropic medicines; (8) long-term commitment; and (9) adequate monitoring in the face of a possibly volatile situation [46].

Monitoring and evaluation are related to the aforementioned repetitive assessment and management cycle. The management cycle moves from problem definition to problem clarification, policy development, decision-making, implementation, and finally, policy evaluation—then cycles back to problem definition [69]. Crucial decisions for the management cycle are the determination of quality aspects to be evaluated (and at what level) and the indicators and criteria to be used. Process, satisfaction, and outcome indicators that are consistent with the predefined objectives should be formulated. They should span both the minimum response during the emergency phase and long-term reconstruction phase. Indicators should be specific, measurable, achievable, relevant, and time-bound (SMART), chosen on the principle of “few but powerful,” and disaggregated by age, gender, and location whenever possible. The Inter Agency Standing Committee (IASC) Reference Group for MHPSS is currently developing a common framework for monitoring and evaluation, including a list of indicators that could help to systematize data collection across programs to allow for more general conclusions. A more rigorous design with a sound theoretical basis at the beginning of any intervention is required to determine whether or not the intervention per se caused the change. An example would be a (cluster) randomized controlled trial and research on moderators and mediators.

### Addressing Gaps in Research

According to Patel [70], the evidence on the etiology and effective treatment of mental health issues is inferior when compared to research on most other chronic and non-communicable diseases, especially in LMIC. A recent review of the evidence resulted in WHO mental health guidelines for conditions and disorders specifically related to stress [16•, 71]. The guideline development group discovered that there is a dearth of rigorous research supporting many of the most commonly used interventions for stress. The group identified recent systematic reviews or commissioned reviews when none were available [16•]. The evidence appeared to be particularly poor for children and adolescents: for 3/11 (PICO) questions asked, no specific recommendations could be made based on existing evidence. For example, there were no specific recommendations for the treatment of acute stress symptoms, secondary non-organic enuresis, bereavement, hyperventilation, insomnia, or the appropriate use of benzodiazepines and antidepressants. The authors agreed that formulation of evidence-based guidelines is complicated by limited knowledge, particularly in LMIC. This includes limited knowledge on the effectiveness of (a) commonly implemented interventions, (b) established evidence-based interventions when used in situations of ongoing adversity, and (c) widely used cultural practices. For example, current popular and common interventions for youth include non-specific counseling, psycho-education, structured recreational and sports activities, and provision of child-friendly spaces. However, research findings for these interventions are not consistent. Moreover, application of the guidelines requires improved knowledge on how to reduce potentially harmful practices that are widely applied. Recommended psychotherapeutic interventions require an approach that balances strengthening the availability and capacity of specialists to train and supervise and shifting to non-specialist delivery of psychotherapy. Strengthening evidence will require collaborative efforts by researchers and practitioners in a bi-directional manner that is mindful of local socio-cultural and health system realities.

Addressing a particular research gap would benefit a public health approach, i.e., knowledge of effective mental health promotion and the prevention of mental disorders. This area of intervention is listed as one of four objectives in the first global action plan for mental health worldwide, the WHO comprehensive mental health action plan 2013–2020. Research on the social determinants of mental health is receiving renewed attention [72], but important gaps in knowledge remain. Research often takes both a life-course perspective and examines risk and protective factors across the life-span. The early childhood period is recognized within this framework as a critical developmental period, since development proceeds sequentially. Later development must build on skills learned earlier in life [73]. Despite this recognition, limited research on risk and protective factors takes place in the early childhood period in LMIC [49]. In particular, research on positive parenting practices and interventions to improve maternal mental health would be greatly beneficial in order to effectively facilitate children receiving a good start in life in this population [74].

Several LMIC, such as the Middle East and those in the Great Lakes area in Africa, are affected by cycles of violence. To disrupt or mitigate these cycles of violence, it is important to study young people’s contribution in this cyclical process in their communities, schools, and families. It is also important to study the transgenerational transmission of violence, in terms of the “mechanisms” or the violence that is transmitted and how this process relates to resilience as a socio-ecological and interactive process across generations. These latter questions indicate the importance of mixed methods research.

## Conclusion

We argued that in (post)conflict areas and disasters, particularly in LMIC, a public health approach is needed to address the mental health of children and adolescents. We provided arguments that a clinical approach will not enable us to bridge the treatment gap in situations of cumulative and often chronic adversity. Given that social conditions, such as a lack of public services, can contribute to the expression and progression of distress and disorders at the individual and population level, there is now a growing interest in syndemics.

We introduced four research and practice strategies that are critical to strengthen a public health approach to mental health for children and adolescents in situations of mass adversity.

First, we argued that theoretical innovation is critical. In combination with socio-ecological theory and syndemics, the concept of transgenerational ecological resilience and systems theory for public health provides a paradigm to reach large populations of youth and families with comprehensive community and prevention-oriented processes. Theoretical innovation would allow more systematic thinking about the collaboration between various sectors, including the health, educational, social, economic, and legal sectors, as well as the implications for adversity and intervention across generations.

Second, we described the multiple phases of setting up a public health program and highlighted the importance of basing programs on detailed needs assessments rather than assumptions of needs. We also emphasized the active involvement of children and young people in the participatory identification of these needs. It is obvious that the process of defining priorities and policies is highly complex and even more so if one tries to divide scarce human and material resources over large populations taking into account both prevention and treatment and the volatility of (post)disaster settings.

Third, we described the importance of a long-term perspective on interventions for children and adolescents in situations of mass adversity and pointed to health systems aspects that promote sustainability such as budgeting and planning for the longer term. We also discussed how program monitoring and evaluation could contribute to improved practice in this field.

Fourth, we identified several specific research questions that warrant attention over the coming years. There are major gaps in knowledge on effective practices for populations in humanitarian settings, particularly for children and adolescents. Formulation of evidence-based guidelines is complicated by limited knowledge regarding the effectiveness of interventions (particularly in situations of ongoing adversity), the effectiveness of widely used cultural practices, and the reduction of potentially harmful practices. We also need more insight into the effects of chronic adversity and poly-victimization of children and the role of children and adolescents in the transgenerational transmission of violence, both in the family and on wider ecological levels. Because children are at the bottom of the social pyramid, they are at the receiving end of many of the obnoxious processes in syndemics.

In summary, we propose a comprehensive multilayered public health approach for children and adolescents affected by humanitarian crises. Such a public health approach would entail theoretical innovation, community-based and participatory program planning that is responsive to social context, a longer-term perspective with a focus on sustainability, and addressing a number of current critical gaps in our knowledge of child and adolescent mental health and well-being.
